# *CYP1A1* gene polymorphisms as a risk factor for pterygium

**Published:** 2010-06-09

**Authors:** Chi-Hsien Young, Yu-Lun Lo, Yi-Yu Tsai, Tung-Sheng Shih, Huei Lee, Ya-Wen Cheng

**Affiliations:** 1 Institute of Medicine, Chung Shan Medical University, Taichung, Taiwan; 2 Institute of Occupational Safety & Health, Taipei, Taiwan; 3 Graduate Institute of Clinical Medical Science, China Medical University, Taichung, Taiwan; 4 Department of Ophthalmology, China Medical University Beigang Hospital, Taichung, Taiwan; 5 Department of Ophthalmology, China Medical University Hospital, Taichung, Taiwan; 6 Graduate Institute of Environmental Health, College of Public Health, China Medical University and Hospital, Taichung, Taiwan; 7 Institute of Medical Molecular Toxicology, Chung Shan Medical University, Taichung, Taiwan; 8 Department of Medical Research, Chung Shan Medical University Hospital, Taichung, Taiwan

## Abstract

**Purpose:**

Both cytochrome P4501A1 (CYP1A1) and glutathione S-transferase M1 (GSTM1) have been demonstrated to be involved in the metabolism of polycyclic aromatic hydrocarbons (PAHs). BaP 7,8-diol 9,10-epoxide (BPDE), an ultimate metabolite of benzo(a)pyrene (BaP), attacks deoxyguanosine to form a BPDE-N2-dG adduct resulting in *p53* gene mutations. Our previous report indicated that BPDE-like DNA adduct levels in pterygium were associated with *CYP1A1* gene polymorphisms. Therefore, we hypothesize that the genetic polymorphisms of CYP1A1 and *GSTM1* increase the risk for pterygium.

**Methods:**

Two hundred-five pterygial specimens and 206 normal controls were collected in this study. For the analysis of *CYP1A1* and *GSTM1* gene polymorphisms, DNA samples were extracted from blood cells and then subjected to restriction fragment length polymorphism and polymerase chain reaction for the determination of mutation and genotype of *CYP1A1* and *GSTM1*.

**Results:**

There was a significant difference between the case and control groups in the *CYP1A1* genotype (p=0.0161) but not in *GSTM1* (p=1.000). The odds ratio of the *CYP1A1* m1/m2 polymorphism was 1.327 (95% CI=0.906–2.079, p=0.135) and the m2/m2 polymorphism was 1.647 (95% CI=1.154–2.350, p=0.006), compared to the m1/m1 wild-type genotype. The *GSTM1* polymorphisms did not have an increased odds ratio compared with the wild type.

**Conclusions:**

In conclusion, a *CYP1A1* polymorphism is correlated with pterygium and might become a marker for the prediction of pterygium susceptibility.

## Introduction

The environmental pollutant, benzo[a]pyrene (BaP), which is one of the polycyclic aromatic hydrocarbons (PAHs), has been found to cause *p53* gene mutations and then lung tumorigenesis. The levels of PAHs in airborne particulates in Taiwan are higher than levels found in other countries, especially levels of BaP, benzo[b]fluoranthrene and benzo[g,h,i]perylene [1,2]. BaP 7,8-diol 9,10-epoxide (BPDE), an ultimate metabolite of BaP, attacks deoxyguanosine to form a BPDE-N2-dG adduct which results in p53 mutations [3]. The *p53* tumor suppressor gene is one of the most commonly mutated genes observed in human tumors. Our previous study indicated that mutations within *p53* were detected in 15.7% of the pterygial samples and deletion mutations were found in the same samples with p53 negative staining and substitution mutations were found in samples with p53 positive staining [4]. However, the cause of *p53* mutations in pterygium is still unclear.

BaP is oxidized by a series of well characterized enzymes such as cytochrome p450 1A1, 2C9 and 3A4 [5,6]. A thymine/cytosine point mutation in the MSPI restriction site of *CYP1A1* has been reported to result in increased enzyme activity [7]. The *CYP1A1* MspI polymorphism has been linked to the susceptibility for smoking-related cancers, such as oral, colon, breast, and lung cancers [8-10]. Not only cytochrome P450 but other enzymes, such as glutathion s-transferase M1 (GSTM1) have been shown to be involved in BaP metabolism [11-13]. *GSTM1* has also been shown to be polymorphic. A deletion is responsible for the existence of a null allele associated with the lack of expression of a functional protein [14,15]. The polymorphic *GSTM1* null genotype has been found in 20%–50% of populations of various ethnic origins, and this genotype has been correlated with the risk for various tobacco-related cancers [16-19]. Therefore, the genetic polymorphisms of *CYP1A1* and *GSTM1* may contribute to BPDE-like DNA adduct formation and pterygium progression.

Our previous report indicated that BPDE-like DNA adducts were detected in pterygium samples and the DNA adduct levels were associated with the genetic polymorphisms of *CYP1A1* [20]. Additionally, the risk of BPDE-like DNA adduct formation for patients with *CYP1A1* m2/m2 and m1/m2 was 9.675 fold higher than that of patients with m1/m1 types. Therefore, we hypothesize that that the genetic polymorphisms of *CYP1A1* and *GSTM1* increase the risk of pterygium.

In this study, we try to analyze the *CYP1A1* and *GSTM1* gene polymorphisms using PCR-RFLP (Polymorphism Chain Reaction-Restriction Fragment Length Polymorphism) and PCR (Polymorphism Chain Reaction) methods in 205 pterygium specimens and 206 controls to understand how *CYP1A1* and *GSTM1* polymorphisms increase the risk of pterygium.

## Methods

### Patients

Pterygial samples were harvested from 205 patients (136 males and 69 females) undergoing pterygium surgery at China Medical University Hospital (Taichung, Taiwan). All of the samples were from patients who had primary pterygium. The age range was 52 to 85 and the average age was 72.4 years old. The control blood samples were collected from patients without pterygium and pinguecula including 126 males and 80 females in the control group (age range from 55 to 75 years, mean of 62 years). There were no significant differences between both groups in age and sex. This study was performed with the approval of the Human Study Committee at China Medical University Hospital.

### Polymorphisms of *CYP1A1* and *GSTM1*

DNA was extracted from the paraffin-embedded pterygium tissues for genetic polymorphism analysis [4]. DNA lysis buffer was applied to lyse the epithelial cells on the slide and then the DNA solution was transferred into eppendorf tubes for traditional proteinase K digestion and phenol-chloroform extraction. Finally, the DNA was precipitated by ethanol with the addition of linear polyacrylamide to increase DNA amounts [21]. Genotyping of the MspI polymorphism of *CYP1A1* was performed by PCR amplification using the primer set of 5′-TAG GAG TCT TGT CTC AGT CCT-3′ and 5′-CAG TGA AGA GGT GTA GCC GCT-3′ [22]. The amplified products were digested with MspI and analyzed by electrophoresis on a 1.5% agarose gel. The MspI restriction site polymorphism resulted in three genotypes: a predominant homozygous m1 allele without the MspI site (genotype m1/m1; C/C), the heterozygote (genotype m1/m2; C/T) and a rare homozygous m2 allele with the MspI site (genotype m2/m2; T/T). Detailed information of the PCR assays used in this study has been described previously [23]. Genotypes of *GSTM1* were determined by the presence or absence of PCR product, according to the method of Groppi et al. [23]. The genotypes of *GSTM1* are defined as present and null type. Two primers, 5′-GAA GGT GGC CTC CTC CTT GG-3′ and 5′-AAT TCT GGA TTG TAG CAG AT-3′, were used for PCR. If samples had no PCR product, the PCR experiment was repeated by adding a set of β-actin (*ACTB*) primers together with *GSTM1* primers, to confirm that the absence of *GSTM1* PCR product represented the null genotype.

### Statistical analysis

Statistical analysis of frequency distributions was done by the χ^2^-test, and the correlations between various genotypes of *CYP1A1* and *GSTM1* from the case and control groups were analyzed by statistical software SPSS 10.0 (SPSS, Chicago, IL). Adjusted odd ratios (ORs) and a 95% confidence interval (95% CI) for various factors of pterygium were evaluated using a multiple logistic regression model.

## Results

### Relationship of *CYP1A1* and *GSTM1* gene polymorphisms and pterygium

To verify the association of risk and the genetic change in the metabolic genes in pterygium development, polymorphisms of *CYP1A1* and *GSTM1* in the pterygium and control groups were analyzed. The results of the genotypes of *CYP1A1* and *GSTM1* in the pterygium and control groups are shown in Table 1. The analysis of the *CYP1A1* polymorphisms in pterygium showed that 68 (33.2%) were homozygous for the m1/m1 genotype, 29 (14.1%) were homozygous for the m2/m2 genotype, and 108 (52.7%) were heterozygous for the m1/m2 genotype. There was a significant difference between the case and control groups in the *CYP1A1* genotype (p=0.016). However, no clear patterns were observed between the pterygium and control groups for significant associations with *GSTM1* polymorphisms.

**Table 1:** Genotype distribution of *CYP1A1* and *GSTM1* genes among pterygium patients and control group.

**Gene**	**Pterygium group (%) [n=205]**	**Control group (%) [n=206]**	**p-value**
*CYP1A1*			
m1/m1	68 (33.2)	89 (43.2)	
m1/m2	108 (52.7)	103 (50.0)	
m2/m2	29 (14.1)	14 (6.8)	0.016
*GSTM1*			
Null	83 (40.5)	84 (40.8)	
Present	122 (59.5)	122 (59.2)	1.000

### The *CYP1A1* gene polymorphism but not *GSTM1* is a risk factor for pterygium

To understand whether polymorphisms of *CYP1A1* and *GSTM1* increased the risk of pterygium development, the different genotypes and the risk of pterygium were compared. The odds ratio of the *CYP1A1* (m1/m2) polymorphism was 1.327 (95% CI=0.906–2.079, p=0.135) and the m2/m2 polymorphism was 1.647 (95% CI=1.154–2.350, p=0.006), compared to the m1/m1 wild-type genotype (Table 2). The *GSTM1* polymorphisms did not increase the odds ratio compared with the wild type (Table 2). The multiple logistic regression analysis showed that the *CYP1A1* genotype is related to the risk of pterygium after an adjustment with *GSTM1* polymorphisms. Subjects who were heterozygous (m1/m2) or homozygous (m2/m2) for the *CYP1A1* polymorphisms appeared to experience a higher risk of pterygium than those who were homozygous for the wild-type allele (m1/m1; OR: 1.553; 95% CI: 1.07–2.290, p=0.037; Table 3). No significance was found in *GSTM1* polymorphisms.

**Table 2:** Risk of pterygium in relation to polymorphisms in genes involved in BaP metabolites in a population-based sample.

**Gene**	**OR**	**95% CI**	**p-value**
*CYP1A1*			
m1/m1	1	-	-
m1/m2	1.327	0.906–2.079	0.135
m2/m2	1.647	1.154–2.350	0.006
*GSTM1*			
Present	1	-	-
Null	1.012	0.683–1.500	0.952

**Table 3:** Multiple logistic regression analysis of *CYP1A1* and *GSTM1* genotypes and the risk of pterygium.

**Variable**	**Groups unfavorable/ favorable**	**OR (95% CI)**	**p-value**
*CYP1A1*	Polymorphism/wild type	1.553 (1.027–2.290)	0.037
*GSTM1*	Null/present type	0.990 (0.666–1.471)	0.959

## Discussion

Our previous study indicated that BPDE-like DNA adducts were detected in pterygium paraffin sections [24]. We also found that *CYP1A1* polymorphisms correlated with the BPDE-like DNA adduct formation in pterygium [20]. Therefore, we considered that not only UV radiation, but also environmental exposure is involved in pterygium pathogenesis. In this study, we analyzed the PAHs metabolic enzymes, *CYP1A1* and *GSTM1*, and their gene polymorphisms in pterygium and compared them with control groups. Our data indicated that the *CYP1A1* polymorphism is a risk factor for pterygium. To our knowledge, this is the first study to analyze the correlation of genetic polymorphisms of *CYP1A1* and *GSTM1* with the risk of pterygium. GST is one of the antioxidant defense enzymes which contributes to the protection against ROS [25,26]. The *GSTM1* null type has been reported to be associated with cutaneous photosensitivity [27,28], so the *GSTM1* null may be associated with photosensitivity of corneal limbal cells. Our previous report indicated that lack of GSTM1 (*GSTM1* null type) contributes to susceptibility of pterygium formation in early onset pterygium but is not associated with late onset pterygium [29]. In this study, we did not find an association between the *GSTM1* polymorphism and the risk of pterygium. Therefore, we suggest that the role of *GSTM1* in pterygium formation is more important in antioxidant defense than in PAH metabolism.

PAH compounds are the products of incomplete combustion of organic material and are thus ubiquitous in the environment (International Agency for Research on Cancer [IARC] World health Organization, 1983). Occupational exposure to PAH-compounds increases the risk of lung, and putatively, other cancers, and is the highest in coke oven workers, other workers in the steel industry, asphalt and bitumen workers, and those exposed to exhaust and working with gasoline. BaP, the well known carcinogen in cigarette smoke, induces G:C-T:A transversions experimentally [30] which are the main mutation types in smoking-related lung cancer [31].

Our previous study showed that BPDE-like DNA adduct levels correlate with *CYP1A1* gene polymorphism in pterygium [20]. An evaluation of DNA adducts induced by BaP and other PAHs is suitable as a risk marker of *p53* mutation. The mutation of the *p53* gene has been noted in more than 50% of all human cancers [32-34]. Additionally, our previous study showed that BPDE-like DNA adducts are indeed detected in pterygium samples and they are minor contributors to the abnormal *p53* gene [24]. In this study, we found that *CYP1A1* with the m1/m2 and m2/m2 genotype has a 1.553 fold risk for pterygium compared with the m1/m1 genotypes. Therefore, we hypothesize that after exposure to environmental PAHs, the *CYP1A1* gene polymorphism may result in high levels of BPDE-like DNA adduct formation contributing to the risk of pterygium formation. The *CYP1A1* MspI polymorphism may be used as a risk factor for pterygium.
